# Real-Time Object Detection for Edge Computing-Based Agricultural Automation: A Case Study Comparing the YOLOX and YOLOv12 Architectures and Their Performance in Potato Harvesting Systems

**DOI:** 10.3390/s25154586

**Published:** 2025-07-24

**Authors:** Joonam Kim, Giryeon Kim, Rena Yoshitoshi, Kenichi Tokuda

**Affiliations:** Research Center for Agricultural Robotics, National Agricultural and Food Research Organization, Tsukuba 3050856, Japan

**Keywords:** object detection, agricultural automation, edge computing, YOLOX, YOLOv12, potato harvesting, data imbalance, real-time processing, Jetson AGX Orin

## Abstract

**Highlights:**

**What are the main findings?**

- YOLOX demonstrated superior throughput (107 vs. 45 FPS, *p* < 0.01) and energy efficiency (0.58 vs. 0.75 J/frame) on the Jetson AGX Orin framework, meeting real-time agricultural automation requirements.
- Despite its lower overall speed, YOLOv12 exhibited significantly better recall for underrepresented soil clods (0.725 vs. 0.512) and improved detection of small objects (0–3000 pixels).

**What is the implication of the main finding?**

- This study provides empirically grounded guidelines for selecting object detection models in agricultural automation: YOLOX for high-speed processing, prioritizing overall throughput and efficiency, and YOLOv12 for applications that require high accuracy on challenging, underrepresented classes where speed is less critical.
- The findings underscore that for edge computing applications, the theoretical architectural advantages of a model and its specific implementation efficiency and resource utilization patterns are critical determinants of real-world performance.

**Abstract:**

In this paper, we presents a case study involving the implementation experience and a methodological framework through a comprehensive comparative analysis of the YOLOX and YOLOv12 object detection models for agricultural automation systems deployed in the Jetson AGX Orin edge computing platform. We examined the architectural differences between the models and their impact on detection capabilities in data-imbalanced potato-harvesting environments. Both models were trained on identical datasets with images capturing potatoes, soil clods, and stones, and their performances were evaluated through 30 independent trials under controlled conditions. Statistical analysis confirmed that YOLOX achieved a significantly higher throughput (107 vs. 45 FPS, *p* < 0.01) and superior energy efficiency (0.58 vs. 0.75 J/frame) than YOLOv12, meeting real-time processing requirements for agricultural automation. Although both models achieved an equivalent overall detection accuracy (F1-score, 0.97), YOLOv12 demonstrated specialized capabilities for challenging classes, achieving 42% higher recall for underrepresented soil clod objects (0.725 vs. 0.512, *p* < 0.01) and superior precision for small objects (0–3000 pixels). Architectural analysis identified a YOLOv12 residual efficient layer aggregation network backbone and area attention mechanism as key enablers of balanced precision–recall characteristics, which were particularly valuable for addressing agricultural data imbalance. However, NVIDIA Nsight profiling revealed implementation inefficiencies in the YOLOv12 multiprocess architecture, which prevented the theoretical advantages from being fully realized in edge computing environments. These findings provide empirically grounded guidelines for model selection in agricultural automation systems, highlighting the critical interplay between architectural design, implementation efficiency, and application-specific requirements.

## 1. Introduction

### 1.1. Research Background

The integration of artificial intelligence technologies into agricultural automation systems emerged as a critical strategy for enhancing productivity in response to the increasing global food demand [1]. Modern precision agriculture leverages variable rate technology to optimize resource application and enhance crop management efficiency [2,3]. The development of robust object detection systems that differentiate between crops and impurities during harvesting is vital for improving both efficiency and product quality [4,5]. Such systems are essential components of automated agricultural machinery, in which real-time performance and high accuracy are equally important requirements [6]. The evolution of agricultural robotics highlighted the importance of robust perception systems capable of operating under diverse field conditions [7,8].

Current agricultural automation systems face major performance limitations that constrain their practical deployment. Existing optical sorting systems typically achieve 85–90% overall accuracy for crop-impurity classification but struggle with small object detection, often exhibiting less than 60% recall for defects smaller than 20 pixels in diameter [9,10,11,12]. These systems demonstrate substantial performance degradation under occlusion and scale disparity, with their accuracy dropping from near-perfect performance for large unobstructed objects to 70–80% for partially occluded or small targets [13]. The processing speed represents another critical constraint, as commercial harvesting operations require at least a 30 FPS throughput to meet processing rates of 12,000–15,000 kg/h [14,15], but many existing implementations struggle to maintain real-time performance under these conditions while ensuring consistent quality control [3]. Recent systematic reviews highlighted the critical importance of balancing computational efficiency with detection accuracy for practical deployment in precision agriculture [16,17].

The agricultural landscape in Japan presents unique challenges compared with those in the United States and Europe, as it is characterized by diverse crop varieties and predominantly mountainous terrain [18,19]. Consequently, harvesting equipment often operates across multiple farms, necessitating the development of standalone artificial intelligence (AI) systems that can be effectively integrated into portable harvesting machinery. These systems must function independently without relying on cloud connectivity and impose major constraints on computational resources and power consumption.

**Figure 1:**
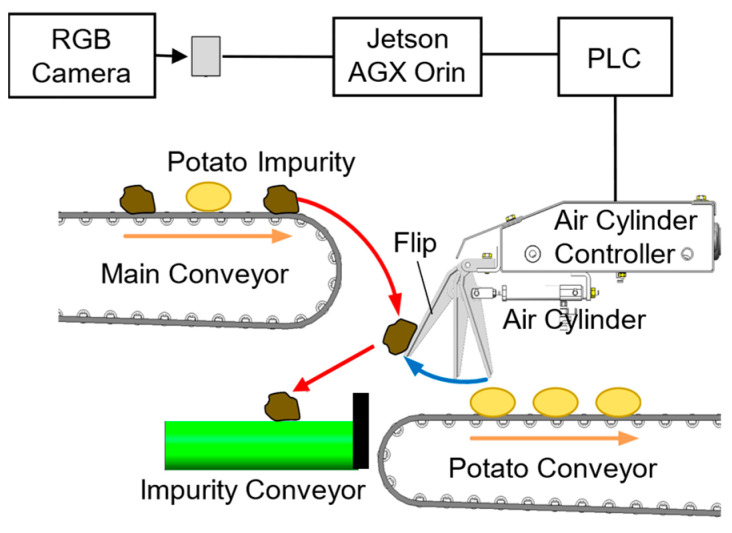
Integrated AI potato harvester system architecture showing RGB camera input, AI edge computer processing, and an PLC-controlled air cylinder for selective impurity removal via an impurity conveyor system.

In the specific context of potato harvesting, automated systems must accurately distinguish between potatoes and various impurities such as soil clods and stones. The architecture of the integrated AI potato harvester system is shown in Figure 1. The system operates through a sequential process, in which objects on the conveyor belt are first captured by an RGB camera. The obtained images are then processed in real time by a Jetson AGX Orin AI edge computer running an object detection model, which classifies each object as either a potato or impurity. Based on this classification, the system either allows potatoes to continue along the main conveyor path or activates an air cylinder controller through a programmable logic controller (PLC) to divert the identified impurities to a separate impurity conveyor. This automated sorting mechanism substantially enhances the processing efficiency while maintaining the strict quality control standards required for agricultural production.

### 1.2. Research Motivation and Objectives

An important challenge in the development of AI systems for agricultural automation is the inherent data imbalance [20]. Owing to commercial quality standards that prioritize marketable produce, training datasets inevitably exhibit imbalances in both object size distribution and class representation [21]. Smaller nonmarketable objects and certain classes of impurities are typically underrepresented in datasets; however, their accurate detection remains crucial for proper sorting [4,5]. Addressing data imbalance through additional data collection is both labor-intensive and time-consuming, making the evaluation of model performance under data imbalance particularly relevant for practical agricultural applications [20,21].

The NVIDIA Jetson AGX Orin platform (Nvidia Corporation, Santa Clara, CA, USA) enables edge computing deployments with comprehensive computational capabilities while maintaining energy efficiency suitable for mobile agricultural equipment [22]. Its Ampere graphics processing unit (GPU) architecture features 2048 Compute Unified Device Architecture (CUDA) cores and an AI performance of up to 275 TOPS. This platform provides the necessary processing power for real-time object detection under field conditions, and its industrial-grade board variant ensures the reliability and robustness required for standalone agricultural AI systems.

The YOLOX model, with its anchor-free design, decoupled head, and simplified optimal transport assignment (SimOTA) labeling strategy, has been widely adopted in agricultural automation systems owing to its license-free status and optimization for high-speed inference [23]. Recent comprehensive reviews documented the accelerating adoption of YOLO architectures across diverse agricultural tasks, including monitoring, surveillance, sensing, automation, and robotic operations, confirming their state-of-the-art performance in agricultural object detection [24,25]. However, concerns emerged regarding its long-term viability following the unfortunate cessation of active development after the passing of its principal developer, risking future support. This development stagnation presents a major risk to industrial applications that require ongoing maintenance and optimization.

By contrast, the YOLOv12 model, released in February 2025 and developed under an integrated Ultralytics research framework, offers promising long-term stability and continued development support [26]. The Ultralytics comprehensive ecosystem and dedicated research team ensure ongoing optimization, bug fixes, and adaptation to emerging hardware platforms. This institutional support provides crucial stability for industrial deployments that require extended operational lifespans. In addition to its development advantages, YOLOv12 introduces novel mechanisms, including a residual efficient layer aggregation network (R-ELAN) backbone and area attention mechanism accelerated by FlashAttention [27], which may offer considerable improvements in both accuracy and processing speed. The contrasting development trajectories of these models—YOLOX uncertain future versus YOLOv12 structured institutional development pathway—represent an important consideration along with their technical capabilities, particularly for agricultural automation systems with expected operational lifespans of several years.

We focus on YOLOX and YOLOv12 as representative and fundamentally distinct architectures rather than pursuing a comprehensive version comparison across all YOLO variants. This selection prioritizes architectural significance to address specific research questions relevant to agricultural automation systems. In detail, YOLOX exemplifies a mature anchor-free paradigm that prioritizes computational efficiency through architecture simplification. Its anchor-free detection with center point regression combined with SimOTA dynamic label assignment represents an optimization-focused approach that allocates computational resources to maximize the throughput while maintaining acceptable accuracy. The decoupled head design further enhances efficiency by allowing independent optimization of classification and regression. In contrast, YOLOv12 embodies an emerging attention-centric paradigm that fundamentally changes the processing and aggregation of spatial information. Its R-ELAN backbone addresses gradient bottlenecks through sophisticated residual aggregation, while the area attention mechanism leverages FlashAttention acceleration for context-aware feature enhancement. This approach represents a shift from efficiency optimization toward intelligent resource allocation based on spatial context and feature importance.

Although the intermediate versions, YOLOv8 and YOLOv9, represent important engineering achievements, they operate within the established anchor-free paradigm without introducing the fundamental computational approaches that define our research focus. YOLOv8 primarily refines the anchor-free approach through enhanced data augmentation and improved label assignment using a task-aligned assigner instead of SimOTA [28]. Similarly, YOLOv9 uses programmable gradient information for training optimization and improving the generalized efficient layer aggregation network backbone [29]. These advances represent valuable optimization refinements within an established paradigm rather than the architectural innovation that distinguishes our comparison. We specifically compare anchor-free optimization with attention-centric approaches under agricultural data imbalance, analyze the computational tradeoffs between established efficient architectures and emerging attention mechanisms on edge platforms, and determine the effects of different label assignment methods on the agricultural class detection performance. YOLOv8 and YOLOv9, while introducing technically sophisticated improvements, fail to address these fundamental paradigmatic aspects because they operate within the same computational framework of YOLOX [29]. Including them in our study would necessarily shift our research focus from architectural paradigm analysis to optimization benchmarking [30], requiring fundamentally different methodologies and evaluation criteria. Instead, out methodological approach aligns with established computer vision research practices, in which architectural analysis focuses on paradigmatic differences rather than exhaustive version comparison. For instance, studies comparing ResNet and the transformer architecture focus on fundamental computational differences rather than including every ResNet variant, enabling deeper architectural insights while maintaining a consistent research focus and efficient resource allocation.

The primary objectives of this research are to

- statistically evaluate the performance of YOLOX and YOLOv12 on the Jetson AGX Orin platform configured based on JetPack version 5.6.3, in terms of speed, accuracy, and resource utilization,
- quantify class- and size-specific performance characteristics with statistical rigor, particularly under data imbalance, and
- establish empirically validated guidelines for model selection in standalone agricultural automation systems, specifically for potato-harvesting applications.

### 1.3. Research Scope and System Requirements

The potato-harvesting system imposes specific performance requirements for object detection. From a system design perspective, the misclassification of potatoes as impurities (false positives) must be strictly limited to less than 1% because such errors directly impact the crop yield. Conversely, the detection rate for impurities (recall) must exceed 60% because subsequent manual quality control processes can address undetected impurities. These asymmetric requirements vary depending on the crop type but represent the established standards for potato-harvesting systems.

This study focuses on a standalone module designed for integration into an actual potato harvester. Both models were trained on identical datasets and optimized using TensorRT 8.5.2 to ensure a fair comparison. Performance evaluation encompasses multiple dimensions, including

- frame rate (FPS) and real-time processing capability,
- class- and size-specific precision and recall metrics under data imbalance,
- performance–power tradeoffs across different clock configurations (AUTO vs. MAX modes), and
- resource utilization patterns and computational efficiency through NVIDIA Nsight 2025.3.1 profiling.

Through this comprehensive comparison across multiple performance dimensions, we aim to determine the practical applicability of both models in edge computing environments and provide empirically grounded guidelines for model selection in standalone agricultural automation systems.

## 2. Materials and Methods

### 2.1. System Architecture and Hardware Setup

The deployment of object detection models in edge computing environments presents distinct challenges beyond theoretical benchmarking [31]. Edge computing emerged as critical for agricultural automation, enabling data processing near the point of generation rather than on centralized servers [32]. This approach reduces latency, conserves bandwidth, and enhances operational autonomy in environments with limited connectivity [33]. Recent advances in edge computing frameworks intended for agricultural applications demonstrated large potential for real-time monitoring and data-driven decision-making in smart farming environments [34].

**Figure 2:**
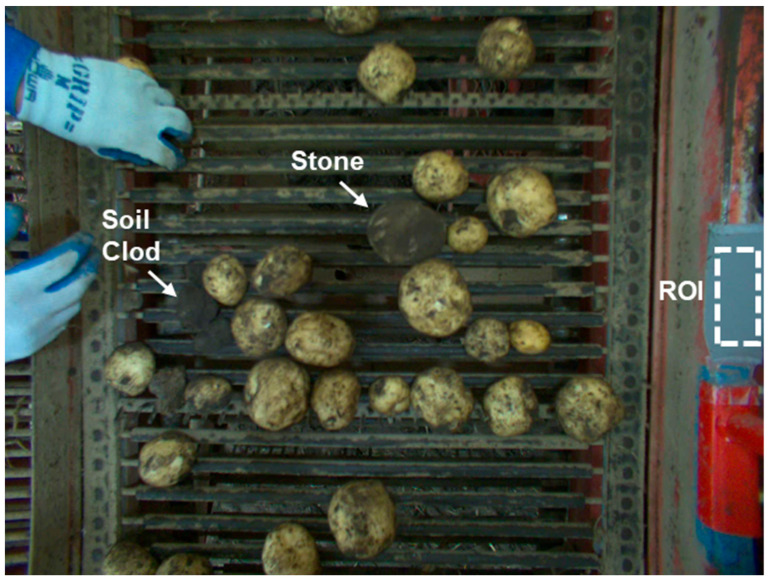
Data collection environment showing conveyor belt transporting potatoes, soil clods, and stones. The region of interest (ROI) indicates the position of the 30% standard gray reference panel used for automatic brightness correction. Representative examples of soil clods and stones are labeled to illustrate the classification challenges addressed in this study.

The images were captured using a Basler acA2040-55uc industrial USB 3.0 camera (Basler, Ahrensburg, Germany) equipped with an IMX265 CMOS sensor (Sony, Tokyo, Japan). The camera provides a 2048 × 1536-pixel resolution (3.2 MP) with a maximum frame rate of 55 FPS. For data collection, images were captured at 5 FPS and subsequently resized to 1280 × 960 pixels for processing and storage. The camera automatic brightness correction system was optimized to maintain optimal illumination for potato surface visualization. As shown in Figure 2, an 18% reflectivity standard gray reference panel was installed next to a conveyor belt with a designated region of interest established on this reference panel for continuous brightness monitoring. The camera automatic exposure time and gain adjustment functions were utilized to maintain a consistent image quality, with the exposure time automatically adjusted within a 1–100 ms range based on the reference panel luminance values. This approach ensured that potato surface characteristics were optimally represented even under varying ambient lighting typical in agricultural field environments.

The NVIDIA Jetson AGX Orin platform, which has an Ampere GPU architecture featuring 2048 CUDA cores and an AI performance of up to 275 TOPS, enables edge computing deployments with comprehensive computational capabilities while maintaining energy efficiency suitable for mobile agricultural equipment. Platforms such as the NVIDIA Jetson series provide GPU acceleration specifically designed for the efficient execution of deep learning models under constrained power budgets [35]. However, as noted in previous research, a substantial gap often exists between the theoretical model performance and real-world implementation, with factors such as memory constraints, thermal limitations, and system integration complexities imposing practical compromises [36].

**Figure 3:**
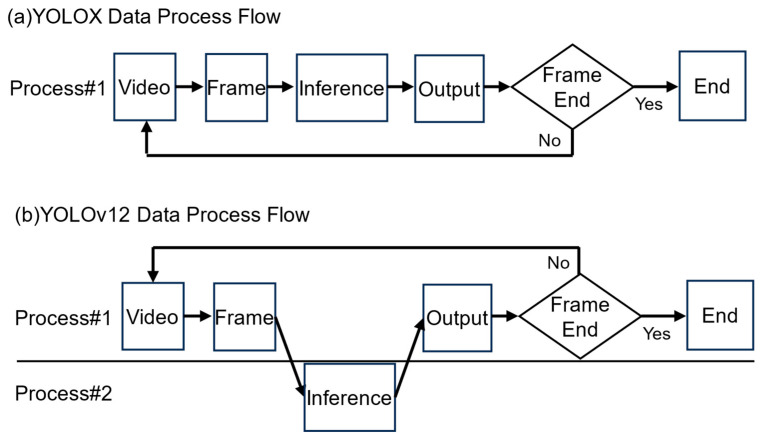
Data process flow comparison between (**a**) YOLOX single-process implementation and (**b**) YOLOv12 multiprocess implementation.

The software architecture for our potato impurity detection system was designed considering these constraints, focusing on real-time performance, reliability, and maintainability. As shown in Figure 3, two distinct approaches were implemented for YOLOX and YOLOv12 owing to their differing requirements and constraints. For YOLOX, a single-process implementation was developed based on the PyTorch (torch-2.1.0+nv23.05-cp38-cp38-linux_aarch64.whl) framework [37] that collected all operations—video capture, frame extraction, inference, and output generation—in a unified process. This approach enabled efficient resource utilization by eliminating interprocess communication (IPC) overhead and facilitating optimized memory access patterns through CUDA stream orchestration [38].

In contrast, the YOLOv12 implementation required a multiprocess architecture, possibly owing to the licensing constraints associated with its framework or specific components. This separation necessitated crossing process boundaries and introduced overhead for synchronization and memory transfers. While providing modular isolation between the inference engine and control logic, this architecture created resource contention and context-switching costs, which affected the overall system performance. However, the Jetson AGX Orin platform utilized a shared memory architecture, which minimized the direct IPC data transfer overhead. Our measurements revealed only a 0.1 ms difference in processing time attributable to the multiprocess design, indicating that the observed performance gap between the models was primarily due to other algorithmic and implementation inefficiencies within YOLOv12 rather than substantial IPC data transfer overhead from the multiprocess architecture on this specific platform.

### 2.2. Object Detection Models

Object detection models drastically evolved over the past decade and are broadly categorized into two- and one-stage methods [39]. Two-stage methods, such as the region-based convolutional neural networks, first generate region proposals and then classify each proposed region, thereby offering high accuracy at the expense of computational efficiency [40]. In contrast, one-stage methods, such as the you only look once (YOLO) series, enable faster inference through a unified network structure, making them particularly suitable for real-time applications in resource-constrained environments. Computer vision techniques are suitable for agricultural applications for crop variety discrimination and quality assessment [41].

**Figure 4:**
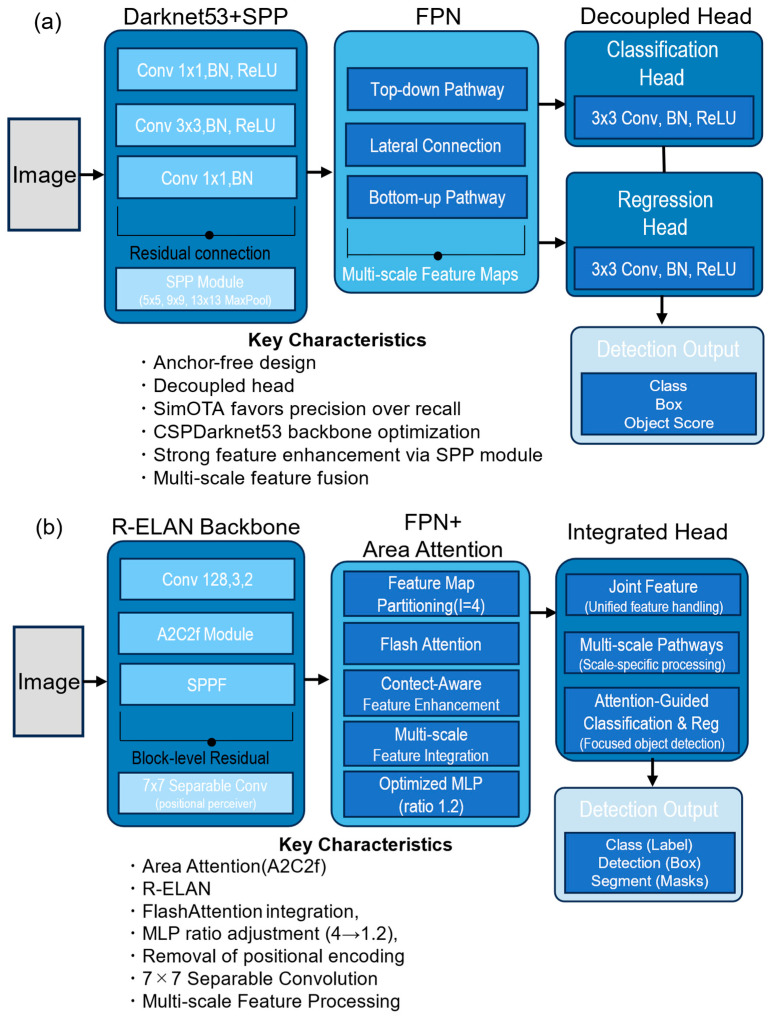
Architecture comparison between (**a**) YOLOX and (**b**) YOLOv12 object detection models showing key differences in backbone networks, neck structures, and head designs that influence detection performance characteristics.

The general architectures of YOLOX and YOLOv12, highlighting their key components from the backbone to the detection head, are described in Figure 4. YOLOX represents a major advancement owing to its anchor-free design, decoupled head-separating classification and regression, and SimOTA labeling strategy. These innovations collectively enhance both accuracy and computational efficiency, explaining the YOLOX widespread adoption in practical applications, including agricultural automation [23]. The anchor-free design eliminates anchor-related hyperparameters, simplifying the detection pipeline, whereas the decoupled head allows for the separate optimization of classification and regression tasks, potentially accelerating convergence.

Recent comprehensive reviews documented the accelerating adoption of the YOLO architecture across diverse agricultural tasks, including monitoring, surveillance, sensing, automation, and robotic operations, confirming its state-of-the-art performance in agricultural object detection [25,42,43]. More recently, YOLOv12 introduced novel architectural components, including an R-ELAN backbone and area attention mechanism accelerated by FlashAttention [26,27]. The area attention mechanism operates within the neck structure by segmenting the input feature maps into distinct spatial regions and applying attention weights to prioritize the computationally critical areas. This attention-centric design first divides feature maps into grid-based regions and then computes attention scores for each region based on feature magnitude and spatial context. FlashAttention optimization is integrated at the kernel level to reduce the memory access overhead during attention computation, enabling efficient processing of the high-resolution feature maps typical in agricultural imaging applications. The R-ELAN backbone aims to address gradient bottleneck issues while enhancing feature fusion through block-level residual connections with scaling techniques, allowing for a more stable gradient flow through deeper networks compared with traditional backbone designs.

### 2.3. Dataset Composition and Characteristics

The dataset used in this study comprised images of potatoes and impurities collected from commercial potato farms in the Hokkaido region of northern Japan. Data collection was performed during actual harvesting, with images captured at 5 FPS as objects moved along the conveyor belt. This capture rate was calculated based on the 0.2 s transit time required for objects to traverse the camera field of view, ensuring complete coverage of all objects while maintaining high data quality. The tractor operated at typical field speeds of 2–3 km/h during harvesting, consistent with common commercial potato harvesting conditions. This real-world collection approach ensured the dataset representativeness for practical agricultural automation deployment, contrasting with static or laboratory-controlled imaging conditions.

A total of 10,000 images were acquired at a resolution of 1280 × 960 pixels. These images were stratified and randomly split, with 8000 (80%), 1000 (10%), and 1000 (10%) images allocated to training, validation, and for testing, respectively, maintaining a consistent class distribution across all subsets. Figure 2 illustrates the data collection environment, showing the diversity of objects encountered during harvesting and demonstrating typical challenges in agricultural object detection, including varying object sizes, soil on the potato surface, and possible visual similarity between soil clods and potatoes that demands expert discernment.

**Figure 5:**
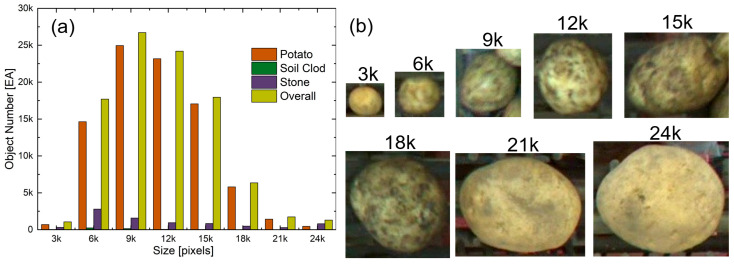
Dataset characteristics showing (**a**) distribution of object sizes across different classes in the dataset, illustrating large imbalance in both class representation and size distribution and (**b**) representative examples of potato samples across different size categories (3–24 × 10^3^ pixels), demonstrating the visual characteristics and scale differences that impact the detection performance.

The dataset encompasses three primary object classes: potatoes, soil clods, and stones. As detailed in Table 1, the dataset exhibits a large class imbalance with potatoes, stones, and soil clods representing 91.0%, 8.4%, and 0.67% of all objects, respectively. Moreover, the class distribution exhibits a large imbalance, with potatoes, soil clods, and stones constituting approximately 60%, 20%, and 20% of all annotated objects, respectively. This distribution reflects real-world harvesting conditions, in which soil clods represent the most challenging class for detection due to their visual similarity to potatoes and severe underrepresentation. As illustrated in Figure 5a, the dataset also displays a substantial imbalance in the object size distribution. For analysis, objects were categorized into eight size ranges based on their pixel area: 0–3, 3–6, 6–9, 9–12, 12–15, 15–18, 18–21, and 21–24 × 10^3^ pixels. Figure 5b shows representative examples of potato samples across these size categories (3–24 × 10^3^ pixels), illustrating the visual characteristics and scale differences that impact the detection performance. The size distribution shows concentration in medium ranges (6–12 × 10^3^ pixels) across all classes, with progressively fewer instances at both smaller and larger scales.

To address the inherent data imbalance, class-weighted loss functions were employed during training, with weights inversely proportional to class frequencies, and a focal loss was implemented to mitigate the impact of easy-to-classify examples that dominated learning. The images were captured under real-world harvesting conditions using the automatic brightness correction from the industrial camera system, ensuring adaptability to varying ambient lighting during actual harvesting. Computer vision approaches demonstrated effectiveness in agricultural classification tasks, particularly for variety discrimination and quality assessment under challenging field conditions [41].

### 2.4. Data Annotation

All images in the dataset were annotated using a semiautomatic annotation approach developed at the National Agriculture and Food Research Organization (NARO). Annotation consisted of two distinct phases to optimize accuracy and efficiency. Initially, 200 images were manually annotated by agricultural experts with extensive experience in potato sorting. These annotations were created following a standardized protocol to establish a high-quality foundation dataset, with manual annotation involving drawing precise bounding boxes around each visible object and assigning an appropriate class label (potato, soil clod, or stone).

Following manual annotation, a semiautomatic annotation support system based on the YOLOv5 architecture was employed to accelerate annotation completion and mitigate the labor-intensive manual annotation. This tool was developed at NARO and selected for its established annotation pipeline stability and reliable preliminary predictions. YOLOv5 was the base architecture, reflecting the tool development history rather than model preference, as annotation was focused on the bounding box accuracy rather than model-specific features.

The semiautomatic approach was implemented primarily to reduce the processing time, providing preliminary bounding box suggestions that substantially reduced the annotation workload while ensuring quality through human oversight. The workflow involved generating initial bounding box proposals through the YOLOv5-based system and comprehensive manual verification and correction by experienced annotators trained in agricultural object classification. All automatically generated annotations were manually verified and corrected by the annotators to ensure quality and eliminate potential model-specific biases. Rigorous human oversight ensured that the final dataset remained completely independent of any model-specific detection biases while dramatically reducing the annotation time from an estimated 500 h or more to approximately 150 h for the complete dataset. The semiautomatic tool served for suggesting preliminary bounding boxes, with 100% manual verification ensuring no YOLOv5-specific detection patterns influenced the training of either the YOLOX or YOLOv12 model.

Specific annotation protocols were established for challenging cases:

- Objects partially outside the image frame or touching image boundaries were excluded from annotation.
- Objects obscured by equipment or hands during data collection were excluded (during system operation, no human hands appear in the field of view).
- For potatoes with heavy soil coverage that hindered visual distinction, classification decisions were made through consultation with experienced agricultural specialists familiar with potato quality grading standards.
- No cases of stones adhered to potato surfaces were encountered in the dataset.
- All ambiguous cases were resolved through consensus among multiple agricultural experts to ensure annotation consistency.

Annotation was conducted under the guidance of experienced agricultural specialists, who provided advice on ambiguous cases and ensured consistency with practical sorting standards. For ambiguous cases where visual distinction based solely on image analysis was challenging, additional consultation with agricultural experts familiar with specific visual characteristics of potatoes, soil clods, and stones was conducted to ensure accurate classification. All classification decisions were made based exclusively on the visual analysis of the captured images without physically handling the objects. The annotation protocol emphasized consistency with established agricultural quality standards used in commercial potato processing facilities to ensure real-world applicability of the trained models.

No additional preprocessing was applied to the images before training because the built-in brightness correction feature of the camera system adequately normalized the input images under various lighting conditions. This approach aligned with the intended deployment scenario, in which the same camera system with identical settings is used. The semiautomatic annotation tool developed at NARO is available for research purposes through institutional collaboration requests, but it is not publicly distributed due to internal registration policies.

### 2.5. Training Configuration and Model Optimization

Both the YOLOX and YOLOv12 models were trained using comparable configurations to ensure a fair evaluation. Training was conducted over 300 epochs for both models. The input images were captured at an original resolution of 1280 × 960 pixels and automatically resized to 640 × 640 pixels internally using the respective frameworks during processing.

For the YOLOX implementation, we utilized the official repository (commit hash, 7c55dad) with its comprehensive hyperparameter configuration. This included three object classes, a depth multiplier of 0.33, width multiplier of 0.50, and single warmup epoch, leveraging the built-in SimOTA labeling strategy. Data augmentation employed mosaic and mix-up transformations with full probability application, HSV color space augmentation, and 50% horizontal flip probability. Training utilized a batch size of 64 with FP16 mixed precision, the AdamW optimizer, and a cosine annealing learning rate schedule at a 640 × 640-pixel input resolution.

For the YOLOv12 implementation, we used the Ultralytics framework (YOLOv12 version 8.3.116) with its specific model architecture. The implementation code used in this study is available at https://github.com/ultralytics/ultralytics/tree/main/ultralytics accessed on 2 March 2025 to ensure complete reproducibility. This implementation utilized a batch size of 8, employing the AdamW optimizer with an initial learning rate of 0.001 and weight decay of 0.0005, thus leveraging the built-in area attention mechanism.

Both models were converted into the TensorRT format with FP16 precision to optimize inference speed and memory usage on the Jetson AGX Orin platform [44]. TensorRT optimization was performed using the following configuration:

- Precision FP16 (half-precision floating point)
- Workspace with 4GB GPU memory
- Batch size of 1 (optimized for real-time single-image inference)
- Input resolution of 640 × 640 pixels
- Dynamic shape optimization disabled for consistent latency
- CUDA optimization enabled with kernel autotuning
- Memory pooling enabled for efficient GPU memory management

Optimization typically required 10–15 min per model on the Jetson AGX Orin platform.

### 2.6. Performance Evaluation

To ensure the statistical robustness and reliability of our comparison, all performance measurements were conducted across 30 independent trials under controlled conditions. Each trial involved complete inference runs on the test set, with metrics recorded for the frame rate, accuracy, precision, recall, and resource utilization patterns.

The frame rates were measured in two configurations: (1) core inference testing measuring the raw processing speed of each model without additional system components and (2) complete system testing evaluating the performance when integrated with all supporting components, including monitoring systems, video backup functionality, and PLC communication interfaces, representing real-world deployment conditions.

Statistical validation was performed using paired *t*-tests to confirm the significance of the performance differences between models, with significance thresholds set at *p* < 0.05. Additionally, the precision and recall metrics were validated using bootstrapping with 1000 iterations to establish 95% confidence intervals (CIs) for all class-specific detection results [45,46].

Resource utilization was analyzed using the NVIDIA Nsight Systems profiling tool to examine the CUDA stream efficiency, memory transfer patterns, and kernel execution characteristics. Power consumption was measured using the integrated power monitoring capabilities of the Jetson AGX Orin development kit, capturing the total system power as well as the component-specific consumption for the GPU, central processing unit (CPU), 5 V system, and 1.8 V always-on power rails. All evaluations were performed using identical video input files for both models to ensure consistent and comparable testing conditions.

All evaluation scripts, statistical analysis codes, and profiling data extraction tools are available in the project repository to ensure complete methodological reproducibility. Although the specific agricultural dataset and trained models cannot be shared owing to institutional policies, the repository includes comprehensive training scripts and evaluation frameworks that allow researchers to reproduce the experimental methodology on their own agricultural datasets. Automated scripts for conducting a 30-trial statistical validation, paired *t*-test analysis, and bootstrapping CI calculations are provided to support rigorous comparative analysis.

## 3. Results

### 3.1. Performance Metric Comparisons

Comprehensive profiling of both models on the Jetson AGX Orin platform revealed significant differences in computational efficiency and detection accuracy. In core inference testing, YOLOX demonstrated exceptional efficiency, achieving 76 FPS in the AUTO mode and 107 FPS in the MAX mode, significantly outperforming YOLOv12, which achieved 16 and 45 FPS, respectively. Core inference testing across 3000 frames (1000 frames × 3 iterations) confirmed these performance differences, with YOLOX consistently achieving a superior energy efficiency of 0.49 J/frame in the AUTO mode compared with YOLOv12 at 0.91 J/frame. When integrated into a complete harvester system with all supporting components (monitoring, video backup, and PLC communication), YOLOX maintained 36 FPS, comfortably exceeding the 30 FPS threshold required for real-time agricultural automation. In contrast, YOLOv12 achieved only 12 FPS under identical conditions, falling significantly below this critical threshold.

The implementation architecture revealed key differences, with YOLOX utilizing an efficient single-process design with 91.1% kernel utilization and an optimized host-to-device memory transfer of 96.1%. YOLOv12, which was constrained by licensing requirements to use a multiprocess implementation, exhibited fragmented execution patterns despite reporting 98.9% kernel utilization with less efficient memory transfer patterns (71.5% host-to-device and 20.2% device-to-device). These architectural differences directly impacted resource utilization, with YOLOX showing balanced and predictable CPU usage patterns compared with the YOLOv12 irregular processing with frequent context switching.

Despite its computational disadvantages, YOLOv12 demonstrated competitive overall detection accuracy with F1-scores of 0.98 for the potato class and 0.88 for impurity classes (overall F1-score, 0.97) compared with the YOLOX overall F1-score of 0.97, highlighting the complex relationship between computational efficiency and detection performance in edge computing environments. To ensure statistical robustness, all performance measurements were repeated across 30 independent trials under controlled conditions. The frame rates listed in Table 2 represent mean values with standard deviations of ±2.1 FPS for YOLOX and ±1.8 FPS for YOLOv12 in core inference testing. The 30 trials yielded consistent results with no observed variation, confirming the reliability and reproducibility of the performance differences between the models. Similarly, the precision and recall were validated using bootstrapping over 1000 iterations, establishing 95% CIs for all class-specific detection results presented in Section 3.3. Bootstrap validation across 1000 iterations confirmed the statistical robustness of the performance metrics of both models. YOLOX achieved an overall F1-score of 97.0% (95% CI, 96.8–97.2%) with precision of 95.8% (95% CI, 95.6–96.0%) and recall of 98.1% (95% CI, 97.9–98.3%). YOLOv12 demonstrated a comparable overall performance (F1-score, 97.4%; 95% CI, 97.2–97.6%) with specialized capabilities including potato detection precision of 99.06% (95% CI, 98.99–99.12%) and soil clod recall of 66.82% (95% CI, 63.21–70.26%), demonstrating consistent performance under agricultural data imbalance.

The computational efficiency gap stemmed from fundamental architectural differences in resource utilization patterns. The YOLOX single-process design with SimOTA labeling created predictable execution patterns optimized for throughput, while the YOLOv12 attention mechanism introduced memory scatter–gather operations and kernel synchronization overhead that fragment GPU execution, despite achieving a superior recall for challenging classes. The performance paradox—YOLOv12 achieving equivalent overall accuracy (F1-score, 0.97) despite computational disadvantages—reveals that attention-centric architectures compensate for efficiency losses through improved detection consistency, particularly for underrepresented classes, which lead the YOLOX quality-based filtering to create systematic disadvantages. This tradeoff has direct economic implications for agricultural deployment, where the YOLOX 36 FPS execution provides operational safety margins, while the YOLOv12 12 FPS establishes a critical bottleneck for real-time harvesting requirements.

### 3.2. Power Efficiency and Resource Utilization

To further examine the practical implications of edge deployment, we conducted detailed power efficiency and resource utilization analyses across multiple operational configurations. Table 2 lists the corresponding results as means ± standard deviations across 30 trials.

Our analysis revealed several remarkable findings regarding power efficiency. YOLOX outperformed YOLOv12 in terms of both processing speed and energy efficiency. When measuring core inference capabilities, YOLOX in the MAX clock configuration achieved 107 FPS while consuming 23.31 W of power, resulting in superior energy efficiency at 0.58 J/frame compared with the 0.75 J/frame of YOLOv12 MAX. Hence, YOLOX MAX was 2.38 times faster than YOLOv12 MAX (45 FPS) while improving energy efficiency by 22% (0.58 vs. 0.75 J/frame). However, in the complete system configuration with all harvester components active, these values decreased to 36 FPS for YOLOX and 12 FPS for YOLOv12, reflecting the additional computational burden of supporting monitoring, image data backup, and PLC communication functionality.

YOLOX demonstrated the expected relations between power settings and energy efficiency, with the AUTO mode achieving superior energy efficiency (0.49 J/frame) despite its lower throughput, and the MAX mode prioritizing performance with acceptable efficiency tradeoffs (0.58 J/frame). YOLOv12 showed the expected pattern, where the MAX mode consumed more power (19.98 vs. 12.34 W) with correspondingly better energy efficiency (0.75 vs. 0.91 J/frame). This phenomenon occurred because the MAX configuration eliminated the computational overhead of dynamic frequency scaling management, allowing the system to complete inference more efficiently and transition to idle states faster, resulting in a lower overall energy consumption per processed frame. In the AUTO mode, the continuous monitoring and adjustment of clock frequencies consumed additional computational resources and introduced processing delays. Additionally, the thermal headroom of the Jetson AGX Orin was sufficient to sustain MAX operations without throttling during our testing intervals, further contributing to its superior efficiency. While YOLOX MAX consumed 35.4% more total power than YOLOX AUTO (23.31 vs. 17.22 W), it delivered 40.8% higher frame rates (107 vs. 76 FPS), resulting in a more complex energy efficiency tradeoff. This suggests that the dynamic clock adjustment overhead in the AUTO mode reduces efficiency, outweighing potential energy savings, particularly for computation-intensive deep learning workloads that benefit from sustained high-frequency operations.

The power distribution across the components revealed architectural differences between the models. YOLOX showed a balanced utilization of both the CPU and GPU resources (CPU, 6.99 W; GPU, 8.87 W in MAX mode), indicating effective parallelization and workload distribution across the available processing units. In contrast, YOLOv12 demonstrated heavier reliance on GPU computation (GPU, 10.13 W; CPU, 2.91 W in MAX mode), suggesting architectural differences in the distribution of processing tasks between CPU and GPU components, with the YOLOv12 attention mechanisms requiring more intensive GPU-specific computations.

### 3.3. Class- and Size-Specific Performance Analysis

While aggregate performance metrics provided a general comparison framework, detailed class-specific analysis revealed critical insights into the architectural impacts on real-world performance. The impurity classes were selected for in-depth analysis for three primary reasons: (1) they represent the greatest detection challenge in potato-harvesting systems, where false negatives directly affect the product quality; (2) they demonstrate the most significant performance variations between the two models, serving as ideal case studies for architectural influence; and (3) they exhibit distinct precision–recall tradeoff patterns that unveil fundamental differences in the balance of detection confidence against recall between the models. Furthermore, the soil class, which is severely underrepresented in the training data, provides a valuable testbed for evaluating the model robustness to data imbalance, which is a common challenge in agricultural applications.

**Figure 6:**
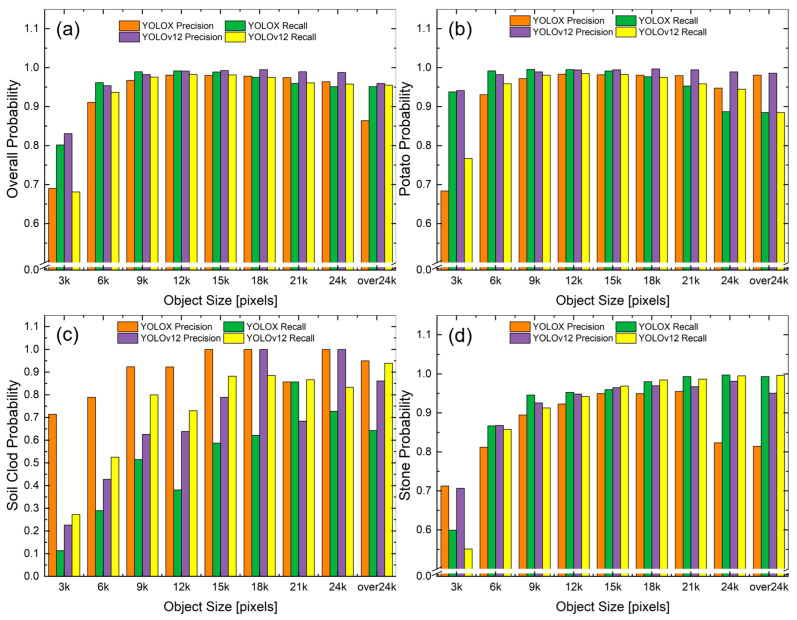
Size-specific precision and recall comparison between YOLOX and YOLOv12 for (**a**) overall performance and (**b**) potato, (**c**) soil clod, and (**d**) stone classes. The statistical confidence was assessed through bootstrap CIs.

Despite its computational disadvantages, YOLOv12 demonstrated competitive detection accuracy across all scenarios. Regarding overall performance, YOLOX achieved an F1-score of 0.97, while YOLOv12 recorded F1-scores of 0.98 for the potato class and 0.88 for the impurity classes (overall F1-score, 0.97). Statistical validation through 1000-iteration bootstrap analysis revealed narrow CIs for YOLOv12 metrics. The overall potato precision of 99.06% (95% CI, 98.99–99.12%) and overall impurity recall of 88.67% (95% CI, 87.94–89.29%) confirmed the statistical significance and reproducibility of the performance characteristics. With nearly identical overall performance (both achieving F1-scores of 0.97), the models demonstrated comparable aggregate detection capabilities, though class-specific analysis revealed distinct performance characteristics and specialized advantages for different detection scenarios. Figure 6 illustrates the size-dependent precision and recall metrics for both models across all object categories and each individual class, revealing significant variations in the detection characteristics. Notably, the overall performance comparison in Figure 6a shows that while both models achieved similar precision across most size categories, YOLOv12 exhibited stronger precision for smaller objects (3–6 × 10^3^ pixels). The class-specific analyses in Figure 6b–d further demonstrate these performance differences across the three primary object classes, with particularly pronounced variations for the soil class.

Class-specific analysis also revealed statistically significant differences in nuanced performance characteristics. For the soil class, YOLOv12 significantly outperformed YOLOX in terms of recall across nearly all size categories, as listed in Table 3. To summarize the precision–recall tradeoffs, the area under the precision–recall curve (PR-AUC) was computed using bootstrap resampling. YOLOX overall PR-AUC was 0.94 (95% CI, 0.92–0.96), while the YOLOv12 PR-AUC was 0.91 (95% CI, 0.89–0.93). For the challenging soil clod class, YOLOv12 achieved a higher PR-AUC (0.81, 95% CI: 0.78–0.84) than YOLOX (0.67; 95% CI, 0.63–0.71). For soil clod objects in the scale of 9–12 × 10^3^ pixels, YOLOv12 and YOLOX achieved recalls of 0.730 and 0.381, respectively, while maintaining a comparable precision (0.639 vs. 0.923). The bootstrap CIs for YOLOv12 soil clod detection (95% CIs, 63.21–70.26% for recall and 52.15–59.13% for precision) demonstrated the statistical reliability of these performance improvements over YOLOX, particularly for challenging impurity classes intended for agricultural automation. Across all size categories for the soil class, YOLOv12 achieved an average recall of 0.725 compared with 0.512 for YOLOX.

Similarly, for small objects (0–3 × 10^3^-pixel scale) across all classes, YOLOv12 demonstrated statistically significant superior precision (0.831 vs. 0.690, *p* < 0.01), as detailed in Table 4, albeit with a lower recall (0.681 vs. 0.802). This size-specific performance difference is particularly important in agricultural applications, where smaller objects are often the most challenging to detect reliably.

The stone class presented a particularly interesting case study on precision–recall tradeoffs between the two models, as listed in Table 5. For the stone class, YOLOv12 outperformed YOLOX in terms of both precision (0.965 vs. 0.950) and recall (0.969 vs. 0.960) for 12–15 × 10^3^-pixel objects, showing more balanced characteristics. This balance is particularly valuable in the context of agricultural automation, where the cost of missing impurities (false negatives) may exceed that of occasional false positives. For the stone class, consistent performance differences were observed between the models across all size categories. Following established statistical validation protocols, the performance differences were evaluated across 30 independent trials. The most pronounced difference was in the 0–3 × 10^3^-pixel category, where the recall of YOLOX (0.599) was consistently higher than that of YOLOv12 (0.551), despite the comparable precisions (0.713 vs. 0.707), even though this difference was not statistically significant.

These detailed performance metrics revealed that data imbalance significantly impacted both models, but to different extents. YOLOX, with its SimOTA labeling strategy focused on high-quality samples, demonstrated a clear preference for precision over recall, particularly for underrepresented classes and sizes. This characteristic manifested most prominently in its performance for the soil clod class, where it achieved high precision but extremely low recall. This operation mode favored high-confidence detection while potentially missing ambiguous cases, thereby minimizing false positives but increasing false negatives. In contrast, the YOLOv12 area attention mechanism and integrated loss function created more balanced precision–recall characteristics across all object classes. By focusing computational resources on critical image regions and leveraging contextual information more effectively, YOLOv12 achieved a substantially higher recall for challenging classes, such as soil clods, with acceptable precision tradeoffs. The performance disparity was the most pronounced for smaller objects (0–3 × 10^3^-pixel category), where the YOLOv12 attention mechanism provided substantial performance improvements despite the inherent difficulties of detecting small objects in complex agricultural environments. These performance differences stemmed directly from architectural design philosophies implemented in each model. YOLOX SimOTA created a precision-biased training regime by systematically filtering samples based on quality thresholds, establishing a feedback loop where challenging samples received progressively less training attention. On the other hand, the YOLOv12 R-ELAN backbone provided robust gradient flow, enabling effective learning from limited samples of underrepresented classes, while the area attention mechanism dynamically allocated computational resources based on spatial importance rather than predefined quality metrics.

### 3.4. Labeling Strategy and Training Dynamics

The labeling strategy, which determined which ground truth objects should be associated with which predictions during training, constituted a critical algorithmic difference between these models. YOLOX implemented a SimOTA, which dynamically assigned labels during training with a focus on high-quality samples [23]. This strategy formulated label assignment as an optimal transport problem solved using a top-*k* approach. Although SimOTA provided computational efficiency and generally improved precision, our in-depth analysis revealed that it created a self-reinforcing mechanism that disproportionately affected underrepresented classes in imbalanced datasets. This mechanism operated via several interconnected processes:

- During training, SimOTA selected only the top-*k* higher-quality samples for each ground truth object based on a combination of classification scores and intersection-over-union values.
- For underrepresented classes such as soil clods, which already suffer from sample scarcity and visual diversity limitations, quality-based filtering further reduced the number of effective training samples.
- This restriction particularly affected objects with irregular shapes, ambiguous boundaries, or background similarity, which characterized the soil clod class.
- This resulted in a feedback loop in which underrepresented classes received less training attention, leading to reduced recall capabilities, specifically for these challenging classes.

This behavior explained the YOLOX performance pattern, that is, higher precision but dramatically lower recall for challenging classes, such as with soil clods, where the recall dropped to a minimum of 0.114 for small instances, while the recall of YOLOv12 was 0.273 for the same category.

YOLOv12 employed the task-aligned assigner integrated within the Ultralytics framework, which fundamentally differed from YOLOX SimOTA. We utilized the default implementations of both models without custom optimization because we aimed to evaluate baseline architectural capabilities for imbalanced agricultural data. Nevertheless, the inherent differences in their assignment philosophies substantially impacted the model performance. The task-aligned assigner considered both classification scores and localization quality through a strategy that maintained more balanced positive sample distribution across classes compared with SimOTA winner-takes-all optimization. This default behavior, combined with the YOLOv12 area attention mechanism, provided inherent compensation for data imbalance without requiring a custom assignment strategy. Our analysis revealed that these architectural default settings resulted in YOLOv12 maintaining higher positive assignment rates for underrepresented classes during training, explaining the observed 42% recall improvement for soil clods despite using unmodified framework implementations. This demonstrated that baseline architectural design choices, rather than custom optimization, could drastically influence the model behavior in imbalanced agricultural data. This balanced assignment strategy appears to be especially effective for small object detection and underrepresented classes, where maintaining sufficient positive samples during training is crucial for agricultural applications with inherent dataset imbalances. Detailed analysis revealed that the YOLOv12 labeling strategy employed graduated quality scoring considering spatial context and attention weights, contrasting with the SimOTA binary quality classification. This nuanced approach prevented complete exclusion of challenging samples, which were assigned a proportionally lower but nonzero training importance. The attention mechanism provided additional context, enabling the model to learn from ambiguous cases that SimOTA would categorically reject, thereby improving the generalization to underrepresented classes and challenging detection scenarios.

### 3.5. NVIDIA Nsight Profiling Analysis

Based on comprehensive NVIDIA Nsight profiling, YOLOX demonstrated more efficient CUDA stream orchestration with operations that were clearly delineated and optimally sequenced. Statistical analysis across 30 profiling sessions confirmed consistent execution patterns with minimal idle periods (*p* < 0.01) [45,46].

To gain deeper insight into the underlying causes of the performance differences, we conducted detailed profiling using NVIDIA Nsight. Table 6 summarizes the key CUDA stream efficiency metrics derived from profiling. Our comprehensive profiling analysis revealed pronounced differences in the execution patterns, memory utilization, and resource management between the two models.

YOLOX demonstrated remarkably efficient CUDA stream orchestration, with operations clearly delineated and optimally sequenced in the profiling data. The NVIDIA Tools Extension Library markers in the YOLOX profile showed distinct operation boundaries and logical groupings, enabling the GPU scheduler to maintain high occupancy. This efficient stream management resulted in a continuous kernel execution pattern with minimal idle periods, achieving 91.1% kernel utilization. The temporal distribution of the kernels showed consistent execution with predictable latencies, indicating a correctly optimized algorithm implementation.

In contrast, YOLOv12 exhibited significantly different execution characteristics despite reporting an impressive aggregate kernel utilization of 98.9%. However, this metric proved to be misleading when examined through a detailed timeline analysis. Although YOLOv12 achieved a high aggregate utilization, the actual execution pattern revealed frequent context switches and suboptimal memory access patterns, which created effective idle periods between productive computation cycles. Kernel distribution analysis revealed highly uneven computational patterns, with the top three kernels—copyPackedKernel (9.5%), permutationKernelPLC3 (7.7%), and DivMulTrs (6.3%)—collectively consuming over 23% of the execution time. These specialized kernels, while enabling YOLOv12 advanced attention mechanisms, created fragmented execution patterns in which the GPU frequently waited for memory operations or kernel synchronization, resulting in a lower effective throughput despite the high nominal utilization rates.

Memory transfer patterns also exhibited substantial differences that directly impacted performance. YOLOX demonstrated highly optimized memory access patterns, with 96.1% of the transfers occurring as efficient host-to-device and minimal device-to-device operations. This pattern indicated a thoughtful memory management strategy that minimized data movement within the GPU memory hierarchy and reduced memory bandwidth contention. By contrast, YOLOv12 showed a less efficient memory utilization profile with 71.5% host-to-device transfers and substantial 20.2% device-to-device operation, suggesting more complex data dependencies and memory reorganization requirements typical of attention-based architectures. This additional memory movement represents a significant overhead in the memory bandwidth-constrained environment of edge computing platforms.

Differences in the implementation architecture also contributed to performance disparities. YOLOv12 implementation was divided into separate inference and control processes, reportedly owing to licensing constraints. Specifically, YOLOv12 multiprocess implementation stemmed from AGPL-3.0 licensing constraints within the Ultralytics framework. The AGPL-3.0 license required complete source code disclosure for any network service software, creating intellectual property concerns for commercial agricultural deployments. Consequently, our implementation isolated AGPL-licensed inference components in separate processes to maintain licensing boundaries while enabling commercial deployment, but this architectural necessity introduced a performance overhead observed in our measurements. The profiling data reveal multiple concurrent Python version 3.8 processes (process identifiers 4746, 4486, 5036, and 5018) for YOLOv12, each competing for system resources. This multiprocess nature led to the task scheduler in the operating system to frequently reallocate CPU time, thus contributing to irregular execution patterns and suboptimal resource allocation, as evidenced by the frequent context switching in the operating system scheduling data. While the Jetson AGX Orin platform shared-memory architecture minimized direct IPC data transfer overhead to 0.1 ms, the management of multiple processes still created resource contention and context-switching costs that affected the performance predictability.

In contrast, the YOLOX unified process model maintained consistent resource access with predictable scheduling patterns. The profiling data exhibit stable CPU utilization, efficient memory residency, and minimal context-switching overhead. This architectural advantage became particularly significant when the system operated under resource contention from the concurrent monitoring, video backup, and PLC communication processes required in the complete agricultural automation system.

The profiling results provide critical insight into the fundamental causes of the performance differences observed between the two models. While YOLOv12 architectural innovations enabled superior detection capabilities for challenging objects, the implementation inefficiencies and computational patterns revealed by our profiling analysis clearly explained its failure to achieve real-time performance in resource-constrained edge computing environments.

Comprehensive profiling revealed three critical differences between model implementations:

- Execution pattern efficiency: YOLOX demonstrated well-orchestrated CUDA streams with 91.1% kernel utilization and minimal idle periods. In contrast, YOLOv12 exhibited fragmented execution with frequent gaps between kernels, despite reporting 98.9% aggregate utilization.
- Memory transfer optimization: YOLOX exhibited efficient memory patterns (96.1% host-to-device) compared with the less optimal YOLOv12 pattern (71.5% host-to-device, 20.2% device-to-device), indicating a higher data movement overhead in YOLOv12.
- Process architecture impact: The YOLOv12 multiprocess implementation, despite its minimal direct IPC data transfer overhead (0.1 ms) on the Jetson platform owing to shared memory, still showed evidence of resource contention and context-switching costs related to managing multiple processes.

These differences, particularly in kernel execution patterns, memory optimization, and the complexities of managing a multiprocess architecture, explained the substantial performance gap observed despite the theoretical advantages of YOLOv12, highlighting the critical importance of implementation efficiency in edge computing deployments.

The complete NVIDIA Nsight profiling data (in format nsys-rep) for both models is available in the project repository at https://github.com/ferrostarkim/YOLOXvsYOLOv12.git accessed on 1 May 2025, enabling researchers to conduct independent verification and extended analysis of the execution characteristics reported in this study.

## 4. Discussion

Recent systematic reviews of deep learning-based object detection in agriculture [47] highlight the critical importance of balancing computational efficiency with detection accuracy for practical deployment.

### 4.1. Architectural Paradigms and Agricultural Applications

Our experimental findings demonstrate that the architectural paradigm selection substantially impacts the agricultural automation performance beyond basic computational metrics. The fundamental difference between efficiency-focused and context-aware approaches is clear from the model handling of data imbalance conditions inherent to agricultural applications. Anchor-free optimization exemplified by YOLOX prioritizes computational efficiency through sophisticated label assignment [30]. However, our results reveal that this approach systematically disadvantages underrepresented classes through quality-based filtering, as evidenced by the dramatically low recall for soil clods. In contrast, the attention-centric paradigm represented by YOLOv12 demonstrates inherent compensation for class imbalance through spatial context awareness, achieving a significantly higher recall for challenging classes while maintaining competitive precision.

### 4.2. Architectural Performance Tradeoffs in Edge Computing

The disparity between YOLOv12 theoretical advantages and practical implementation is a key finding of this study. The YOLOv12 R-ELAN backbone and area attention mechanism demonstrate superior feature representation, particularly for challenging classes such as soil clods and small objects. However, these architectural benefits are significantly hindered by implementation inefficiencies in edge computing environments. While the direct IPC overhead from YOLOv12 multiprocess architecture is minimal on the Jetson AGX Orin shared memory system (0.1 ms), the implementation of the attention mechanism creates fragmented GPU execution patterns, and the overall management of multiple processes appears to contribute to reduced effective resource utilization and context-switching costs. Our research provides a nuanced understanding that YOLOv12 speed limitations stem primarily from these implementation and algorithmic execution challenges rather than inherent architectural deficiencies or substantial IPC data transfer costs on this platform, preventing its theoretical advantages from being fully realized in practical edge deployments. This highlights the critical importance of considering both algorithmic innovation and implementation efficiency when selecting models for edge computing applications.

### 4.3. Data Imbalance and Model Selection in Agricultural Contexts

The interplay between data imbalance and model architecture has critical implications for agricultural automation systems. YOLOX SimOTA label assignment creates compounding effects on underrepresented classes through quality-based filtering, which further reduces the number of effective training samples for already scarce classes, such as soil clods. This mechanism systematically disadvantages objects with irregular shapes or ambiguous boundaries, creating a feedback loop in which underperforming classes receive less training attention.

In contrast, the YOLOv12 components act as countermeasures to data imbalance. The area attention mechanism distributes the computational focus to critical regions regardless of the initial confidence, whereas the R-ELAN robust feature extraction captures subtle visual patterns even with limited examples. For agricultural applications, where balanced datasets are practically impossible to obtain owing to commercial quality standards and collection constraints, this resilience represents a major advantage that extends beyond raw performance metrics.

### 4.4. Application-Specific Model Selection Guidelines

Agricultural automation systems require carefully balanced performance characteristics tailored to operational requirements.

#### 4.4.1. Conveyor-Based Agricultural Automation Systems

For standalone potato harvesting systems operating at conveyor-belt speeds up to 0.6 m/s, the processing speed requirements are determined by mechanical timing constraints rather than throughput optimization. Unlike factory-based conveyor systems in which higher frame rates translates to increased production capacity, the throughput of agricultural harvesters is determined by the harvester movement speed (typically 2–3 km/h) across fields [48]. The critical 30 FPS threshold ensures proper PLC-controlled timing of the sorting mechanism, derived from system-level timing analysis of factors including the object free-fall speed, high-speed pneumatic cylinder response time, and network communication delays between detection and actuation components. Below this threshold, timing mismatches cause systematic misclassification regardless of the detection accuracy.

- YOLOX deployment advantages:
- Reliable 36 FPS operation providing a necessary safety margin for mechanical timing.
- Consistent processing speed across varying field conditions.
- Energy-efficient operation suitable for mobile agricultural equipment.
- Stable resource utilization patterns compatible with standalone systems.
- YOLOv12 deployment considerations:
- Superior feature extraction from compressed inputs (640 × 640 pixel resizing) through Area Attention mechanism.
- Enhanced detection accuracy for challenging classes (42% higher soil clod recall: 0.725 vs. 0.512).
- Better precision for small objects (0–3 × 10^3^ pixels: 0.831 vs. 0.690).
- Current implementation limitations (12 FPS) preventing deployment despite architectural advantages.
  - Future optimization potential through native C++/TensorRT implementation and framework maturation.
- System-specific trade-offs: The balance between computational efficiency and detection accuracy must consider non-negotiable mechanical constraints. While the YOLOv12’s attention-centric architecture demonstrates clear advantages for challenging agricultural detection scenarios, current efficiency limitations in implementation render YOLOX the practical selection for real-time standalone systems requiring precise mechanical timing.

For applications that prioritize real-time processing, such as high-speed conveyor sorting, the YOLOX computational efficiency and stable resource utilization are more appropriate. Its precision-focused characteristics align well with scenarios in which false positives directly impact yield, and auxiliary mechanical filtering can address small object challenges.

Conversely, for quality control systems where the detection accuracy for challenging classes takes precedence over processing speed, YOLOv12’s architectural advantages become valuable despite implementation inefficiencies. Its superior recall for the soil class (average recall of 0.725 vs. 0.512) and balanced precision–recall characteristics for small objects demonstrate clear benefits in specialized detection contexts.

Architectural differences also influence precision–recall tradeoffs, with direct practical implications. The YOLOX decoupled head and SimOTA, focusing on high-quality samples, create precision-favored detection profiles, minimizing false positives but potentially missing ambiguous cases. The YOLOv12 integrated approach produced more balanced characteristics, particularly when the missing impurities (false negatives) exceed the cost of occasional false positives.

#### 4.4.2. Operational Environment Considerations

- Field Conditions: Japanese agricultural environments present unique deployment challenges including variable power availability, extreme temperature variations, and limited connectivity. The YOLOX lower power consumption of YOLOX(0.49–0.58 J/frame vs. 0.75–0.91 J/frame for YOLOv12) provides operational advantages in resource-constrained mobile equipment.
- Integration Complexity: The single-process architecture of YOLOX simplifies integration with existing PLC systems and reduces potential points of failure in field deployments. On the other hand, YOLOv12’s multiprocess requirements, while providing modular benefits, introduce additional complexity for agricultural technicians with limited computing expertise.

### 4.5. Implementation Efficiency and System Integration

NVIDIA Nsight profiling reveals that the implementation architecture significantly affects the practical performance beyond algorithmic design. YOLOX demonstrates efficient CUDA stream orchestration (91.1% kernel utilization) and optimized memory patterns (96.1% host-to-device transfers), whereas YOLOv12 exhibits fragmented execution despite its higher aggregate utilization statistics. These differences highlight the critical importance of considering both architectural innovation and implementation efficiency when selecting models for edge computing applications. The consistent energy efficiency advantage of YOLOX (0.49–0.58 J/frame) over YOLOv12 (0.75–0.91 J/frame) across all tested configurations demonstrates that practical deployment considerations often outweigh theoretical architectural advances in resource-constrained environments.

These findings have broad implications for the design of mechatronic systems for agricultural automation. The performance of the vision algorithm directly affects the subsequent mechanical actuation timing and system-level integration complexity [7]. Current agricultural robotics research emphasizes the critical importance of integrating advanced perception systems with mechanical components to optimize the automation performance [8,49]. Recent comprehensive surveys highlighted the growing importance of imitation learning and advanced machine learning in agricultural robotics, demonstrating the critical role of intelligent vision systems in advancing sustainable agriculture automation [50]. In fact, the performance of vision algorithms directly affects the subsequent mechanical actuation timing and system-level integration complexity. The YOLOX stable performance enables wide operating windows for actuation mechanisms, whereas the YOLOv12 enhanced detection capabilities justify additional computational resources in accuracy-critical applications. With an improvement in the YOLOv12 implementation efficiency through optimization and licensing constraint resolution, its superior data-imbalance handling capability can make it a preferred choice for agricultural applications, even with moderate performance requirements. This emphasizes the importance of tracking both algorithmic advancement and practical optimization maturity when selecting models for long-term agricultural automation deployment.

## 5. Conclusions

The presented case study showcase the implementation experience and establishes a methodological framework through a comprehensive comparative analysis of the YOLOX and YOLOv12 models for object detection in agricultural automation implemented on the Jetson AGX Orin edge computing platform. Through a systematic examination of architectural differences and performance characteristics, in addition to empirically validated guidelines for model selection in potato-harvesting systems, we establish empirically grounded implementation guidance rather than definitive model selection prescriptions. These results align with recent advances in agricultural automation [51] that emphasize the importance of lightweight models for resource-constrained environments. Existing research has demonstrated the effectiveness of optimized YOLO variants intended for agricultural applications, achieving superior performance while maintaining computational efficiency suitable for edge deployment [52].

### 5.1. Key Findings

- **Performance characteristics:** YOLOX demonstrated superior computational efficiency (107 vs. 45 FPS in core inference, 36 vs. 12 FPS in complete system) and energy efficiency (0.58 vs. 0.75 J/frame in MAX mode configurations). Statistical analysis across 30 trials confirmed significant performance differences (*p* < 0.01), with YOLOX achieving superior energy efficiency at 0.58 J/frame compared to the 0.75 J/frame of YOLOv12 in the MAX mode configurations. Both models demonstrated comparable overall detection accuracy (F1-scores of 0.97 for both YOLOX and YOLOv12) with distinct performance characteristics. Bootstrap validation over 1000 iterations established 95% CIs confirming the YOLOX superior computational efficiency alongside competitive accuracy, while YOLOv12 achieved specialized performance advantages including 99.06% potato detection precision (CI, 98.99–99.12%) and 66.82% soil clod recall (CI, 63.21–70.26%), providing statistically robust implementation experience for agricultural automation systems.
- Detection tradeoffs: While YOLOX favored precision-focused detection suitable for minimizing false positives, YOLOv12 demonstrated superior recall for challenging classes such as soil clods (average recall, 0.725 vs. 0.512) and small objects (0–3 × 10^3^ pixels), indicating better resilience to agricultural data imbalance.
- Architectural impact: The YOLOv12 R-ELAN backbone and area attention mechanism provide enhanced feature-representation capabilities, which are particularly valuable for underrepresented classes. However, implementation inefficiencies prevented these theoretical advantages from being fully realized in edge computing environments. Inefficiency sources include fragmented kernel execution, complex memory patterns, and multiprocess architecture complexities, despite the minimal direct IPC overhead due to shared memory.
- Data-imbalance resilience: The YOLOX SimOTA labeling created compounding effects on already underrepresented classes, whereas the YOLOv12 attention mechanism demonstrated inherent compensation for data imbalance, a critical advantage in agricultural applications where balanced datasets are practically impossible to obtain.

### 5.2. Practical Implementation Experience

This case study demonstrates that for real-time potato-harvesting systems requiring a processing performance of at least 30 FPS, YOLOX is the optimal choice owing to its exceptional computational efficiency (107 vs. 45 FPS) while maintaining equivalent overall detection accuracy (F1-score, 0.97) and stable resource utilization compared with YOLOv12. The implementation experience reveals the gap between YOLOv12 theoretical advantages and practical implementation highlights the importance of considering both architectural design and optimization maturity in model selection for agricultural automation systems.

Additionally, this case study establishes a methodological framework for evaluating object detection models in agricultural edge computing environments, encompassing performance evaluation protocols with 30-trial statistical validation and bootstrap confidence intervals, multi-dimensional assessment covering speed, accuracy, energy efficiency, and class-specific performance analysis, implementation characterization through NVIDIA Nsight profiling for understanding performance bottlenecks, and agricultural context considerations including data imbalance handling and real-world deployment constraints. Rather than providing definitive recommendations, this study provides guidance researchers and practitioners to make informed decisions based on specific agricultural automation requirements and constraints.

### 5.3. Limitations and Future Research Directions

This case study has various limitations that should be considered when interpreting the results and their broader applicability. The research was limited to comparison between YOLOX and YOLOv12 without including intermediate YOLO versions (e.g., YOLOv8 and YOLOv9). While these models represent important developments in object detection, we focus on paradigmatic architectural differences between anchor-free optimization and attention-centric approaches. This research focus provides fundamental insights into agricultural automation system design rather than incremental performance comparisons within the same computational framework.

To address the implementation bias introduced by the YOLOv12 multiprocess architecture, future work should investigate single-process implementations using native TensorRT C++ deployment and eliminating framework-level constraints. Alternative approaches include containerized deployment strategies that may minimize the process management overhead while maintaining licensing compliance. Additionally, exploring the YOLOv12 performance with optimized CUDA kernels intended for agricultural edge computing may reveal its true architectural potential independent of current framework limitations.

While data collection was geographically limited to potato farms in the Hokkaido region, a preliminary analysis suggested broader applicability than initially anticipated. In harvester environments, specific features of potato varieties become largely irrelevant due to substantial soil adhesion that masks surface characteristics and natural shape variation within varieties that often exceed inter-variety differences. The most critical detection parameters appear to be environmental conditions—particularly soil moisture affecting adhesion patterns and color variation—rather than geographical or varietal factors. This suggests that the model performance may primarily depend on environmental calibration rather than region-specific training, potentially enabling broader deployment with appropriate parameter adjustments. Comprehensive multiregional validation remains a research priority for future work beyond the architectural comparison scope of this study.

The Jetson AGX Orin platform is the optimal—and arguably only viable—edge computing solution currently available for real-time agricultural automation requiring a processing performance of at least 30 FPS. CPU-based edge platforms fail to fundamentally achieve the required inference speeds because they lack dedicated AI acceleration hardware. Alternative GPU-enabled edge platforms, such as the Google Coral tensor processing unit, are incompatible with the YOLO architecture, while the Intel Neural Compute Stick (Santa Clara, CA, USA) provides insufficient computational power for real-time YOLO inference. Full-scale GPU solutions are impractical for agricultural harvesters due to size, weight, power consumption, and environmental durability constraints. Rather than representing a limitation, this platform selection reflects the current technological reality of edge computing for agricultural automation.

The YOLOv12 multiprocess implementation required due to framework integration constraints introduces implementation-specific limitations that may not reflect the model’s optimal performance potential. While our analysis accurately characterized current deployment realities, future framework optimizations may drastically alter the performance landscape. The licensing and framework integration challenges identified in this study represent current limitations rather than fundamental architectural constraints. Methodological limitations include the reliance on semiautomatic annotation with potential model-specific biases despite human verification. The inherent dataset class imbalance, while representative of real-world agricultural conditions, may have influenced training dynamics in ways that are difficult to fully characterize or generalize across different agricultural applications.

Future research should explore diverse promising directions to address these limitations and extend the findings. Investigation of optimization strategies intended to improve the YOLOv12 implementation efficiency on edge platforms may unlock its theoretical architectural advantages while maintaining practical deployment viability. This includes exploring native CUDA implementations, framework-agnostic deployment strategies, and attention mechanism-based optimization tailored for resource-constrained environments. SimOTA adaptation strategies for imbalanced agricultural data represent another critical research direction. Developing labeling modifications that better accommodate underrepresented classes without sacrificing computational efficiency may notably improve the YOLOX performance in agricultural applications. This might include hybrid labeling strategies that combine the SimOTA efficiency with attention mechanisms for challenging object classes.

Cross-platform performance evaluation across different edge computing architectures may strengthen the generalizability of model selection guidelines. Expanding the evaluation to include diverse crops, environmental conditions, and geographical regions can provide more comprehensive guidance for agricultural automation deployments. Future research should explore integration with variable rate technologies to enable dynamic optimization of agricultural operations based on real-time detection results [37,47]. Integration studies examining the interaction of different object detection models with mechanical sorting systems under varying conveyor belt speeds and environmental conditions may enhance understanding for practical deployment.

The investigation of ensemble approaches combining the computational efficiency of YOLOX with the attention-centric detection capabilities of YOLOv12 represents an intriguing direction for performance optimization across different agricultural scenarios. Such hybrid systems will be able to dynamically select detection strategies based on real-time analysis of scene complexity and processing requirements.

While we utilized the PyTorch framework to ensure a fair architectural comparison, the choice between development and production frameworks requires strategic consideration. PyTorch enables rapid prototyping and reliable comparative analysis essential for research objectives, and the framework overhead does not invalidate the fundamental architectural performance differences observed. Native C++/TensorRT implementations may provide performance improvements for final deployment but represent post-research engineering optimization rather than fundamental architectural evaluation requirements. The primary research objective—evaluating attention-centric versus efficiency-focused paradigms for agricultural data imbalance—is addressed through current solutions, and the PyTorch-based YOLOX implementation already exceeds real-time requirements (36 FPS, surpassing the 30 FPS threshold) for practical agricultural automation deployment. For critical agricultural automation systems requiring a consistent inference latency below 10 ms, a direct CUDA C++ implementation may provide 20–30% performance improvements and reduced power consumption. However, this optimization path requires massive development resources and specialized CUDA programming expertise, which may not be readily available in typical agricultural research and development teams.

### 5.4. Limitations and Broader Applicability

While the presented case study focuses on potato harvesting in Hokkaido, Japan, the methodological framework and architectural insights provide valuable implementation guidance for similar agricultural automation applications. Our comparison are limited to YOLOX and YOLOv12 without including intermediate YOLO versions (e.g., YOLOv8 and YOLOv9). Hence, the focus on paradigmatic architectural differences between anchor-free optimization and attention-centric approaches provides fundamental insights into agricultural automation system design rather than incremental performance comparisons within the same computational framework.

The comparative analysis methodology and performance evaluation framework established in this case study can be adapted to other crop types and geographical regions. Nevertheless comprehensive multiregional validation remains a research priority for future work beyond the architectural comparison scope of this study.

### 5.5. Future Research Directions

Building upon this implementation experience detailed in this study, future research should explore diverse promising directions to address current limitations and extend the findings. For instance, investigation of optimization strategies intended to improve the YOLOv12 implementation efficiency on edge platforms may unlock its theoretical architectural advantages while maintaining practical deployment viability. This includes exploring native CUDA implementations, framework-agnostic deployment strategies, and attention mechanism-based optimization tailored for resource-constrained environments.

As implementation optimization techniques continue to evolve and constraints are addressed, the YOLOv12 architectural innovations demonstrated in the presented case study may be further leveraged in practical edge computing deployments, particularly for applications where data imbalance resilience is critical for agricultural automation success.

In conclusion, this case study provides contributions regarding both specific implementation experience and a generalizable methodological framework, likely enabling the agricultural automation community to make evidence-based decisions while acknowledging the inherent context-dependency of optimal model selection in diverse agricultural environments.

## Figures and Tables

**Table 1:** Dataset characteristics by class and size.

Size Category (×10^3^ pixels)	Potatoes	Soil Clods	Stones	Total
0–3	708	44	327	1079
3–6	14,648	259	2800	17,707
6–9	24,961	165	1592	26,718
9–12	23,183	63	956	24,202
12–15	17,066	46	846	17,958
15–18	5817	37	500	6354
18–21	1444	7	300	1751
21–24	470	33	813	1316
Total	88,297	654	8134	97,085

**Table 2:** Power efficiency and performance comparison on the Jetson AGX Orin platform.

Model Configuration	Frame Rate (FPS)	GPU Power (W)	CPU Power (W)	5 V System Power (W)	1.8 V Always-On Power (W)	Total Power (W)	Processing Time (s)	Total Energy (J)	Energy Efficiency (J/frame)
YOLOX AUTO	76	5.34	4.80	5.69	1.39	17.22	85	1463.7	0.49
YOLOX MAX	107	8.87	6.99	6.00	1.45	23.31	75	1748.3	0.58
YOLOv12 AUTO	16	4.99	0.92	5.37	1.06	12.34	221	2727.14	0.91
YOLOv12 MAX	45	10.13	2.91	5.70	1.24	19.98	112	2237.8	0.75

**Table 3:** Performance metrics for soil clod class across different size categories.

Size Category (×10^3^ pixels)	YOLOX			YOLOv12		
	Precision	Recall	F1-Score	Precision	Recall	F1-Score
0–3	0.714	0.114	0.196	0.226	0.273	0.247
3–6	0.790	0.290	0.424	0.428	0.525	0.471
6–9	0.924	0.515	0.662	0.626	0.800	0.702
9–12	0.923	0.381	0.539	0.639	0.730	0.681
12–15	1.000	0.587	0.740	0.789	0.882	0.833
15–18	1.000	0.622	0.767	1.000	0.886	0.939
18–21	0.857	0.857	0.857	0.684	0.867	0.765
21–24	1.000	0.727	0.842	1.000	0.833	0.909

**Table 4:** Overall performance metrics for small objects (0–3 × 10^3^ pixels).

Metric	YOLOX	YOLOv12
Precision	0.690	0.831
Recall	0.802	0.681
F1-score	0.742	0.749
No. true positives	865	742
No. false positives	388	151
No. false negatives	214	347

**Table 5:** Performance metrics for stone class across different size categories.

Size Category (×10^3^ pixels)	YOLOX			YOLOv12		
	Precision	Recall	F1-Score	Precision	Recall	F1-Score
0–3	0.713	0.599	0.651	0.707	0.551	0.619
3–6	0.812	0.867	0.838	0.868	0.858	0.863
6–9	0.895	0.946	0.920	0.926	0.913	0.919
9–12	0.923	0.953	0.938	0.948	0.942	0.945
12–15	0.950	0.960	0.955	0.965	0.969	0.967
15–18	0.950	0.980	0.965	0.970	0.985	0.977
18–21	0.955	0.993	0.974	0.967	0.987	0.977
21–24	0.823	0.998	0.902	0.951	0.996	0.973

**Table 6:** CUDA stream efficiency summary.

Model	Kernel Utilization (%)	Host-to-Device Transfer (%)	Device-to-Device Transfer (%)	Average Execution Time (ms)
YOLOX	91.1	96.1	2.3	8.4
YOLOv12	98.9	71.5	20.2	12.7

## Data Availability

Due to institutional data protection policies, neither the agricultural dataset nor the trained model weights used in this study are publicly available. However, the complete implementation code for model training, inference pipeline, performance evaluation metrics, and statistical analysis scripts are available at https://github.com/ferrostarkim/YOLOXvsYOLOv12.git accessed on 1 May 2025. Additionally, the original NVIDIA Nsight profiling data (in format nsys-rep) used for execution analysis are provided as compressed files in the repository. The provided training scripts and evaluation framework enable full methodological reproducibility on external agricultural datasets. All code and profiling data support independent verification of the experimental methodology and comparative analysis framework presented in this study.
